# Sharp Increase in Eating Disorders among University Students since the COVID-19 Pandemic

**DOI:** 10.3390/nu13103415

**Published:** 2021-09-28

**Authors:** Marie-Pierre Tavolacci, Joel Ladner, Pierre Déchelotte

**Affiliations:** 1 Clinical Investigation Center 1404, CHU Rouen, U 1073, Normandie University, UNIROUEN, F 76000 Rouen, France; 2 Department of Epidemiology and Health Promotion, CHU Rouen, U 1073, Normandie University, UNIROUEN, F 76000 Rouen, France; joel.ladner@chu-rouen.fr; 3 Department of Nutrition, CHU Rouen, U 1073, Normandie University, UNIROUEN, F 76000 Rouen, France; pierre.dechelotte@chu-rouen.fr

**Keywords:** COVID-19, eating disorders, obesity, university student

## Abstract

The COVID-19 pandemic has caused stress as well as modified physical activity and eating habits among university students. The objectives were to identify the changes in body mass index (BMI) and eating disorders among university students between 2009 and 2021. Between 2009 and 2021, five repeated cross-sectional studies were conducted among university students who filled in an anonymous online self-questionnaire. Age, gender, and BMI were recorded, and the SCOFF questionnaire was used for ED screening which, in combination with BMI, allows to identify the four broad categories of ED with the Expali algorithm. With the five studies, 8981 university students were included in total. Obesity steadily increased between 2009 and 2021, for both men and women. The prevalence of ED was stable between 2009 and 2018 and significantly increased from 31.8% in 2018 to 51.8% in 2021 for women (*p* _trend_ < 0.0001), and from 13.0% in 2009 to 31.3% in 2021 for men (*p* _trend_ < 0.0001). All types of ED increased significantly between 2009 and 2021, except for restrictive ED among men. These results indicate for the first time a significant increase in ED prevalence among students since the COVID-19 pandemic. Initiatives to reinforce early screening of ED to implement targeted interventions in the student population are urgently needed.

## 1. Introduction

Eating disorders (ED) among university students have become a major public health challenge [1]. The college years coincide with the typical age of onset of EDs with a significant concern among college students, especially women and stressed students [2]. On 11 March 2020, Word Health Organization declared the COVID-19 pandemic [3]. Fear of contracting the virus combined with the implementation of public health measures including stay-at-home orders, lockdowns, and closures of universities caused a decrease in physical activity [4] and a modification in eating habits among students which could change their body mass index [5]. Multiple lockdowns, self-isolation, food insecurity, and e-learning implementation due to the COVID-19 pandemic have been associated with anxiety and depression symptoms among students [6], leading to increased risk of developing or worsening ED [7]. However, these studies were limited because of a lack of comparison data collected before the pandemic, which made it difficult to attribute college students’ ED to the pandemic. It is therefore relevant to determine the impact of the COVID-19 pandemic on students when it comes to BMI and the prevalence of ED.

## 2. Methods

Between 2009 and 2021, five repeated cross-sectional studies (Ta Santé en un Clic study [8]) were conducted in the first quarter of 2009, 2012, 2015, 2018, and in May 2021 among students of the University of Rouen Normandy. For each survey, 30,000 students of a French University were invited to participate in the study by email. Volunteering students filled in an anonymous online self-questionnaire. Age, gender, and self-reported height and weight were recorded. Body mass index (BMI) was calculated using the standard formula (BMI weight [kg]/height [m^2^]) and was classified as: underweight (BMI < 18.5), normal weight (BMI between 18.5 and 24.9), overweight (BMI between 25.0 and 29.9), and obese (BMI > 30). The French five-item validated the Sick, Control, One Stone, Fat, Food (SCOFF) questionnaire which was used for ED screening. This, in combination with BMI, allows the identification of four broad categories of ED: restrictive, bulimic, hyperphagic, and other unspecified ED using the validated Expali algorithm [9]. Restrictive disorders include anorexia nervosa, restrictive food intake disorder, and atypical anorexia nervosa; bulimic disorders include bulimia nervosa or bulimia nervosa of low frequency or duration; hyperphagic disorders include binge eating disorders and binge eating disorder of low frequency or duration; and other eating disorders include purging disorder, and night Eating Syndrome and any other ED.

### Statistical Analysis

Qualitative variables were summarized by percentage and a 95% confidence interval (95% CI) as well as continuous variables by mean with standard deviation (SD). The main potential collected confunders factors were sex and age. To limit these biases, the analyses was stratified by gender and students over 25 years old were excluded. The Cochran–Armitage test was used to calculate the *p*_trend_. A *p* value below 0.05 was considered to be significant. The analysis was conducted using XLSTAT by Addinsoft, Paris, France 2020.3.1.

## 3. Results

**Figure 1:**
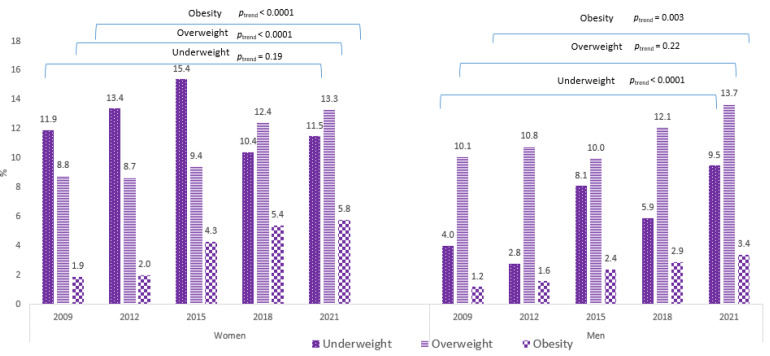
Underweightness, overweightness, and obesity among university students according to the gender, 2009–2021 (*N* = 8981).

Among the 30,000 students addressed in each study, the convenience sample included 1872 university students in 2009, 1217 in 2012, 1730 in 2015, 1478 in 2018, and 3357 in 2021. The participation rate ranged from 4% in 2012 to 11% in 2021. The total sample was 68.7% women with a mean age of 20.4 year (SD = 1.8). From 2009 to 2021, the prevalence of underweight was higher among women than among men (12.1% and 6.3%; *p* < 0.0001, respectively). The same goes for obesity (4.3% and 2.4%; *p* < 0.0001, respectively). There was no difference regarding overweight between genders (*p* = 0.59). Underweightness, overweightness, and obesity by gender for each of the five cross-sectional studies are displayed in Figure 1. For women, overweightness and obesity significantly increased between 2009 and 2021 (from 8.8 to 13.3%; *p*_trend_ < 0.0001, and 1.9 to 5.8%; *p*_trend_ < 0.0001, respectively). For men, underweightness and obesity significantly increased (from 4.0 to 9.5%; *p*_trend_ < 0.0001, and from 1.2 to 3.4%; *p* = 0.003, respectively).

**Figure 2:**
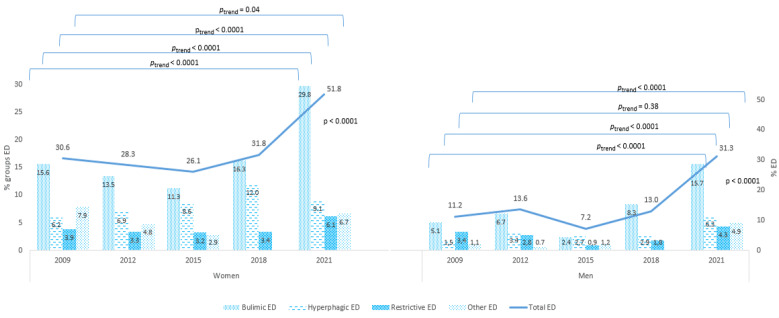
Distribution of ED categories among university students according to the gender 2009–2021 (*N* = 8981). ED: Eating Disorder.

Prevalent EDs and categories of EDs are displayed in Figure 2 by gender and for each of the five cross-sectional studies. For women, EDs increased significantly from 30.6% to 95% CI (28.0–33.2%) in 2009, and from 51.8% to 95% CI (49.8–53.7%) in 2021 (*p*_trend_ < 0.0001), with no changes between 2009 and 2018 (*p*_trend_ = 0.92). A similar trend of ED was observed among the men, from 11.3% to 95% CI (8.7–13.6%) in 2009, and from to 31.3% to 95% CI (28.1–34.4%) in 2021 (*p*_trend_ < 0.0001), with a stability between 2009 and 2018 (*p*_trend_ = 0.81). Regarding ED categories, bulimic ED was the most frequent ED regardless of gender and the year of the study, with a prevalence in 2021 of 29.8% among women and 15.7% among men. All types of EDs increased significantly between 2009 and 2021 except for restrictive EDs among men.

## 4. Discussion

To our knowledge, these results are the first to indicate a significant increase in ED prevalence among students during the COVID-19 pandemic. Our study among students at a university in France, repeated five times over 12 years, shows a steady increase in obesity among women and men, which has increased threefold during this period. This is consistent with pathways by which this pandemic may be exacerbated by ED risk. As mentioned by Rodgers et al., social restrictions may deprive individuals of social support and adaptive coping strategies; an increase the exposure to harmful eating and appearance-related media, particularly on social media; and may increase fears of contagion and emotional distress [10]. In addition, elevated rates of stress and negativity may be affected due to the pandemic, and social isolation may also contribute to increasing risk. Overweightness has also increased to a lesser extent in women. This study highlights how the COVID-19 pandemic impacted several eating behaviors, especially among women. While this context may have increased home cooking, other unhealthy changes occurred, such as increased snacking and weight gain This increase in weight may certainly be due to a decrease in physical activity [4]. This can be explained by the COVID-19 pandemic. Indeed, the constant perception of threat and experience of emotional distress during the COVID-19 pandemic has been associated with adopting coping behaviors that allow people to respond to stress and uncertainty, which are not always associated with healthy behaviors. Using comfort food as a response to negative emotions (i.e., emotional eating) is well known as a coping mechanism in the face of acute stressors [11]. Salazar et al. highlighted that the COVID-19 pandemic could have an effect on comfort food consumption over time with the role of emotional distress [12]. Many college students experienced a shift in food security after the onset of COVID-19, which may be related to the closure of college campuses, changes in housing situation, or changes in employment.

The prevalence of EDs was stable between 2009 and 2018, and increased sharply in 2021 for both women and men. Flaudias et al. showed that individuals with pre-existing eating concerns knew a higher risk for developing problematic eating behaviors during an adverse event—such as the COVID-19 pandemic—and that stress related to the lockdown and social distancing was associated with a greater likelihood of reporting both binge eating and diets [7]. Bulimic ED is the most frequent ED regardless of the year and affected more than one in four female students in 2021. This increase in bulimia was observed both before and during the pandemic. The lockdown was required at home for 24 h a day, seven days per week, with no escape from distancing oneself from food at home [13]. In our study, anorexic disorders doubled for both men and women. The negative emotional effects of lockdown and social limitations are likely to be accentuated for many anorexia nervosa sufferers who are already isolated, both emotionally and physically [14].

Caution is advised for the following reasons. First, the selection bias with a convenience sample had more women than men in the university population. Second, the information bias, which used the SCOFF test and the ExpaliTM algorithm, stated inaccurate clinical diagnoses [9], and the self-reporting of collected data meant that BMI could be underestimated [15]. However, the standardized method of the repeated survey and the stratified analysis by gender limited the bias and allowed a comparison during the 2009–2021 period.

From a public health perspective, this finding underlines the need to extensively screen for ED in students population and identify vulnerable individuals at risk of ED because of excess concerns about food, body shape, weight, and limited resources to cope with the stress induced by the COVID-19 pandemic. Potentially effective strategies in this context may include online-based diagnostic and prevention/coaching interventions. Initiatives to implement early screening of ED and targeted interventions in this high-risk population are urgently needed.

## Data Availability

Data are available on request.
